# A dynamical climate-driven malaria early warning system evaluated in Uganda, Rwanda and Malawi

**DOI:** 10.1186/1475-2875-13-S1-P99

**Published:** 2014-09-22

**Authors:** Adrian M Tompkins, Francesca Di Giuseppe, Felipe Colon-Gonzalez, James Chirombo, Jean Pierre Bizimana, Didacus Namanya

**Affiliations:** 1 Abdus Salam International Centre for Theoretical Physics (ICTP), Trieste, Italy; 2 European Centre for Medium Range Weather Forecasts (ECMWF), Reading, UK; 3 University of North Carolina Project-Malawi, Lilongwe, Malawi; 4 Malawi Ministry of Health, Lilongwe, Malawi; 5 Department of Geography, College of Science and Technology, University of Rwanda, Kigali, Rwanda; 6 Uganda Ministry of Health, Kampala, Uganda

## Background

Malaria is a climate-sensitive disease with a significant socio-economic impact. As monthly and seasonal dynamical climate prediction systems have improved their skill in the tropics over recent years, there is now the potential to use these forecasts to drive dynamical malaria modelling systems to provide early warnings of climate-related transmission hazards in holo and hyper endemic settings.

## Materials and methods

A new pilot dynamical malaria prediction system was introduced. Multiple temperature and precipitation forecasts from the ECMWF monthly and seasonal prediction systems were used to drive the spatially explicit, dynamical malaria model VECTRI that accounts for population density and climate to produce forecasts of up to 4 months ahead. The malaria forecasts were started from realistic initial conditions derived from climate observations. The parameter predicted is the logarithm of the entomological inoculation rate (EIR). Forecasts are made on a grid mesh with a spatial resolution of 25km, but are then aggregated at the administrative district level, and normalized to be evaluated using normalized district level crude incidence data or sentinel site cases (suspected or confirmed) for three countries: Uganda, Rwanda and Malawi. The forecasts are evaluated over a period of approximately a decade, depending on the length of the data record available.

## Results

The results show that for a number of districts in which interventions have been limited over the evaluation period, the forecasts were statistically significantly correlated with observed malaria outcomes, with the forecasts predicting the years of anomalous transmission and the sub-seasonal anomalies well. This was true for districts of both low and higher endemicity. However, for the majority of locations, significance levels were lower. The potential reasons for this are numerous, including incorrect climate forecasts, errors or missing physics (e.g. lack of immunity) in the malaria model, inaccuracies in the cases data itself, and interventions that have reduced prevalence and transmission risk as a function of time. We demonstrate, using confirmed sentinel site data, that data inaccuracies in the district statistics can be significant. Interestingly, the analysis and forecast system appear to indicate that the reduction in malaria prevalence in Rwanda over the past decade has been achieved against a backdrop of increasing transmission hazard due to climate. We conclude by discussing the weaknesses in the system that require addressing and the next steps to pilot this system in an operational context.

